# Redeveloping antigen detection kits for the diagnosis of rat hepatitis E virus

**DOI:** 10.1128/jcm.00710-23

**Published:** 2023-12-01

**Authors:** Zihao Chen, Guanghui Li, Jianwen Situ, Zhiyong Li, Shaoqi Guo, Yang Huang, Shusheng Wu, Zimin Tang, Guiping Wen, Siling Wang, Mujin Fang, Yingbin Wang, Hai Yu, Siddharth Sridhar, Zizheng Zheng, Ningshao Xia

**Affiliations:** 1 State Key Laboratory of Vaccines for Infectious Diseases, Xiang An Biomedicine Laboratory, Department of Laboratory Medicine, School of Public Health, Xiamen University, Xiamen, Fujian, China; 2 National Institute of Diagnostics and Vaccine Development in Infectious Diseases, State Key Laboratory of Molecular Vaccinology and Molecular Diagnostics, National Innovation Platform for Industry-Education Integration in Vaccine Research, NMPA Key Laboratory for Research and Evaluation of Infectious Disease Diagnostic Technology, School of Public Health, School of Life Sciences, Xiamen University, Xiamen, Fujian, China; 3 Department of Microbiology, School of Clinical Medicine, Li Ka Shing Faculty of Medicine, The University of Hong Kong, Pokfulam, Hong Kong, China; 4 The First Affiliated Hospital of Xiamen University, Xiamen University, Xiamen, Fujian, China; 5 United Diagnostic and Research Center for Clinical Genetics, Women and Children’s Hospital, School of Medicine & School of Public Health, Xiamen University, Xiamen, Fujian, China; 6 State Key Laboratory of Emerging Infectious Diseases, The University of Hong Kong, Hong Kong, China; 7 Carol Yu Centre for Infection, The University of Hong Kong, Hong Kong, China; 8 Research Unit of Frontier Technology of Structural Vaccinology, Chinese Academy of Medical Sciences, Xiamen, Fujian, China; Boston Children's Hospital, Boston, Massachusetts, USA

**Keywords:** *Rocahepevirus ratti*, *Paslahepevirus balayani*, rat hepatitis E virus, antigen diagnosis

## Abstract

The emergence of *Rocahepevirus ratti* [species HEV *ratti* (*r* HEV)] as a causative agent of hepatitis E in humans presents a new potential threat to global public health. The *R. ratti* genotype 1 (*r*-1 HEV) variant only shares 50%–60% genomic identity with *Paslahepevirus balayani* [species HEV *balayani* (*b* HEV)] variants, which are the main causes of hepatitis E infection in humans. Here, we report antigen diagnoses for *r*-1 HEV and *b* HEV using an enzymatic immunoassay (EIA) method. We detected recombinant virus-like particles protein (HEV 239) of *r* HEV and *b* HEV using a collection of hepatitis E virus (HEV)-specific monoclonal antibodies. Two optimal candidates, the capture antibody P#1-H4 and the detection antibodies C145 (P#1-H4*/C145^#^) and C158 (P#1-H4*/C158^#^), were selected to detect antigen in infected rat samples and *r*-1 HEV- or *b* HEV-infected human clinical samples. The two candidates showed similar diagnostic efficacy to the Wantai HEV antigen kit in *b* HEV-infected clinical samples. Genomic divergence resulted in low diagnostic efficacy of the Wantai HEV antigen kit (0%, 0 of 10) for detecting *r*-1 HEV infection. Compared with the P#1-H4*/C145^#^ candidate (80%, 8 of 10), the P#1-H4*/C158^#^ candidate had excellent diagnostic efficacy in *r*-1 HEV-infected clinical samples (100%, 10 of 10). The two candidates bind to a discrete antigenic site that is highly conserved across *r* HEV and *b* HEV. P#1-H4*/C145^#^ and P#1-H4*/C158^#^ are efficacious candidate antibody combinations for rat HEV antigen detection.

## INTRODUCTION

Hepatitis E virus (HEV) is the leading cause of enterically transmitted acute hepatitis worldwide (1, 2). HEV infection is usually self-limiting but can lead to chronic HEV infection in immunosuppressed patients. The Hepeviridae family of HEV variants comprises two subfamilies: Orthohepevirinae (terrestrial mammals and birds) and Parahepevirinae (fish) (3). The Orthohepevirinae subfamily includes four genera: *Paslahepevirus*, *Avihepevirus*, *Rocahepevirus*, and *Chirohepevirus*. The *Paslahepevirus* genus consists of two species, *Paslahepevirus alci* and *Paslahepevirus balayani* [species HEV *balayani* (*b* HEV)], the latter comprising eight genotypes, of which five [*balayani*-1 (*b*-1) to *balayani*-4 (*b*-4) and *balayani*-7 (*b*-7)] are pathogenic to humans (4, 5). The *Rocahepevirus* genus also contains two species, *Rocahepevirus eothenomi* and *Rocahepevirus ratti*, which consists of four genotypes [*ratti*-1 (*r*-1) to *ratti*-4].

Rats serve as a natural reservoir of *R. ratti* genotype 1 (*r*-1 HEV) strains, which were previously thought to only infect rodents. However, indirect serological studies in 2011 and 2016 identified *R. ratti* infections in forestry workers and febrile inpatients in Germany and Vietnam, respectively (6, 7). In 2018, Sridhar et al. (8) reported a liver transplant recipient in Hong Kong to be infected with hepatitis caused by *r*-1 HEV, and this marked the first confirmed instance of its ability to transmit to humans. Subsequent studies demonstrated that *r*-1 HEV infections in Hong Kong, Central Africa, Spain, and France were a cause of acute and chronic hepatitis E (HE) in humans (9 – 13). These data provide sufficient evidence of the risk of emerging *r*-1 HEV zoonotic transmission globally.

*b* HEV shares only 50%–60% full-genomic identity with generic *r*-1 HEV (8, 14). The divergence of antigenicity between *r-1* HEV and *b* HEV raises questions about the diagnostic efficacy of the commercial Wantai HEV antigen kit, which uses a *b* HEV-antigen-targeted antibody for *r*-1 HEV-infected patients (15). A previous study found that amino acid (a.a.) divergence resulted in complete loss of sensitivity for a commercial HEV antigen enzymatic immunoassay (EIA) kit, while divergent genomic identity resulted in missed *r*-1 HEV infections when *b* HEV-based reverse-transcription PCR (RT‒PCR) assays were used (8, 16). This study aimed to address this gap in available diagnostics for *r*-1 HEV infection by developing an antigen EIA.

## MATERIALS AND METHODS

### HEV p239 construction, expression, and purification

The open reading frame 2 (*ORF2*) gene sequences of 11 *b* HEV and species HEV *ratti* (*r* HEV) variants were downloaded from GenBank. p239 is a truncated form of the capsid protein that self-assembles into virus-like particles (VLPs) and mimics the conformation of natural HEV particles. Genes encoding *b*-1 HEV to *b*-8 HEV and *b*-3 rabbit (*b*-3ra) p239 (a.a. 368–606), except *b*-2 and *b-7*, were derived from GenBank (accession nos. D11092, AF082843, JF915746, AB573435, AB602441, KX387867, and JX109834, respectively). Gene fragments encoding *r*-1 HEV p239 (a.a. 357–597) and *r*-2 HEV p239 (a.a. 369–609), corresponding to *b* HEV p239, were derived from GenBank nos. KM516906, MG813927, and AB890001 (8, 14). Genes encoding the mutant p239 proteins bearing Ala substitutions were obtained previously (17). All HEV p239 genes were cloned and inserted into pTO–T7 plasmids (18). Recombinant *b* HEV and *r* HEV p239 proteins were subsequently overexpressed in *E. coli*. ER2566. Proteins were harvested from inclusion bodies and treated by dissolution in 4-M urea. Renatured proteins were obtained in 20-mM phosphate-buffered saline (PBS, pH = 7.4) by gradient dialysis as previously reported (16, 19). Renatured proteins mixed with nonreducing and reducing 6× loading buffer were subjected to SurePAGE, Bis-tris, 8%–16% gradient SDS-polyacrylamide gel electrophoresis (GenScript). Renatured proteins were stained using Coomassie blue staining according to standard laboratory protocols.

### Monoclonal antibodies and binding ability

One hundred and four monoclonal antibodies (mAbs) from humans immunized with HEV 239 vaccine and 50 mAbs from humans infected by *b* HEV were selected for further binding testing (20). The binding abilities of mAbs in *b* HEV p239 and *r* HEV p239 were assessed in EIAs. Purified HEV p239 proteins were coated onto the wells of 96-well microplates at 100 ng/well and incubated overnight at 4°C. Wells were blocked with 0.5% (wt/vol) casein in PBS at 37°C for 2 h. The mAbs diluted to 20 µg/mL were serially diluted five fold into the wells and incubated at 37°C for 30 min. After five washes with PBST, wells were incubated with horseradish peroxidase (HRP)-conjugated goat anti-human IgG secondary antibodies (Thermo Scientific, 1:5,000) at 37°C for 30 min. The microplates were washed five times and incubated with 100 µL of tetramethylbenzidine (TMB) substrate. The reaction was stopped by the addition of 50 µL of 2-M H_2_SO_4_ after incubation for 15 min at 37°C. The OD_450 nm_ was measured with a reference wavelength of OD_630 nm_.

### Patient samples

Archived sera from patients with *b* HEV infection in Dongtai, Jiangsu Province (China), Hong Kong (China) and blood donors in Xiamen, Fujian Province (China) were retrieved (21 – 26). Serum samples from patients with *b* HEV infection tested positive for *b* HEV RNA by RT‒PCR and tested positive with the Wantai HEV antigen kit. Serum samples from blood donors tested negative for *b* HEV RNA by RT‒PCR and tested negative with the Wantai HEV antigen kit. Serum samples from patients with *r*-1 HEV infection were obtained from infected patients in Hong Kong as described previously (8, 11, 12).

### Antigen assays for the sandwich EIA

Each of the nine mAbs from humans immunized with the HEV 239 vaccine or infected with *b* HEV had similar binding ability to *b* HEV p239 and *r* HEV p239 proteins. A sandwich EIA format was established to assess the ability of antibody pairs to bind to *b* HEV p239 and *r* HEV p239. Purified mAbs were coated onto wells of 96-well microplates at 500 ng/well. *b* HEV p239 or *r* HEV p239 in 100 µL of PBS was added to the wells and incubated at 37°C for 60 min without washing. Wells were incubated with 100 µL of HRP-conjugated mAbs at 37°C for 30 min. The microplates were washed five times and the incubated with 100 µL of TMB substrate. The reaction was stopped by the addition of 50 µL of 2-M H_2_SO_4_ after incubation for 15 min at 37°C. The OD_450 nm_ was measured at a reference wavelength of OD_630 nm_. Sera obtained from rats infected with *r*-1 HEV were obtained as described previously (16, 27). Rat samples and human samples were analyzed using the same protocol as the Wantai HEV antigen kit, and 50 µL of serum was added into the wells containing 20 µL of diluent and incubated at 37°C for 60 min without washing.

### MAb blocking assays

Purified HEV p239 proteins were coated onto the wells of 96-well microplates at 100 ng/well. Four mAbs, P#1-H4, C145, C158, and 12F12, diluted to 200 µg/mL, were serially diluted twofold in wells and incubated at 37°C for 30 min without washing. The wells were incubated with HRP-conjugated mAbs as a blocking group (OD_blocking_) at 37°C for 30 min. PBS was added to the wells as a control (OD_control_). HRP-conjugated mAbs were used at selected dilutions that resulted in OD readings of 1–2 in the presence of PBS. Data were processed and transformed using the formula (OD_control_ − OD_blocking_)/OD_control_ to calculate the blocking rate.

## RESULTS

### Characteristic analyses of the E2s domain a.a. sequences from *r* HEV and *b* HEV strains

**Fig 1:**
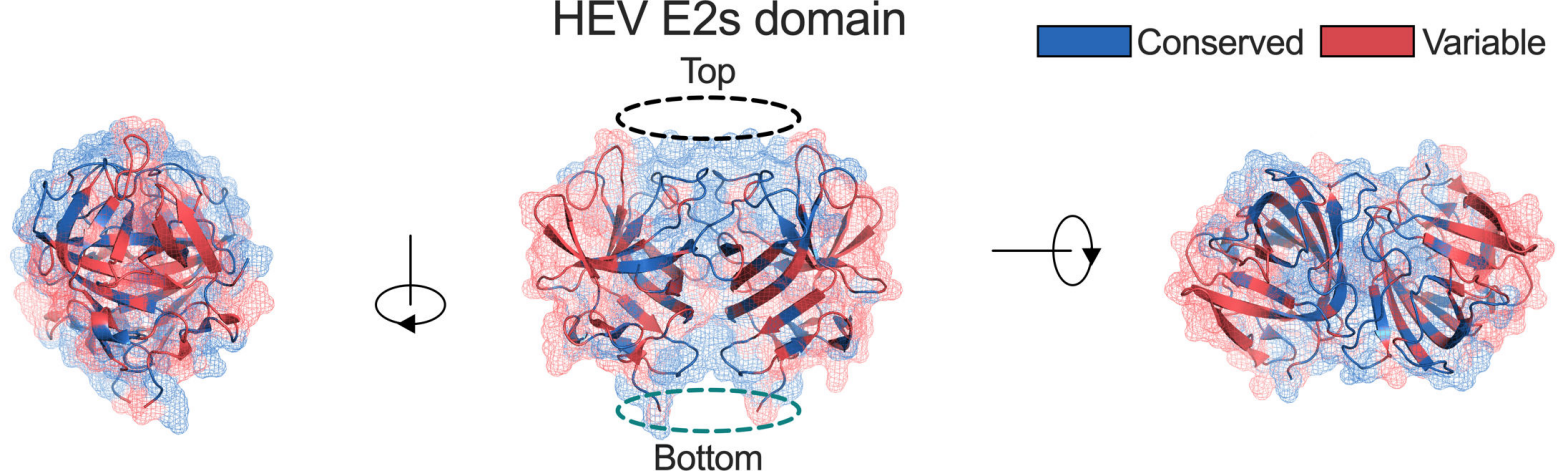
Evolutionary conservation of the E2s domain between *r*-1 HEV and *b*-1 HEV. The a.a. sequences of the E2s domains of *r*-1 HEV LCK-3110 strain (GenBank no. MG813927) and *b*-1 HEV Xinjiang strain (GenBank no. D11092) were analyzed. The E2s domain crystal structure (PDB no. 4PLK) of *b*-1 HEV is depicted in mesh and cartoon representation and colored from red (variable) to blue (conserved). Abbreviations: a.a., amino acid; *b*-1 HEV, species HEV *balayani*-1; HEV, hepatitis E virus; *r*-1 HEV, *R. ratti* genotype 1.

The HEV *ORF2* gene encodes the HEV capsid protein. The *T* = 1 HEV capsid protein is composed of a protruding domain (P domain, a.a. 452–606), a middle domain (M domain, a.a. 318–451) and a shell domain (S domain, a.a. 118–317). The E2s domain (a.a. 459–606) that is located within the P domain of the ORF2 capsid protein includes immune-dominant epitopes for binding and neutralization (3). The commercial Wantai HEV antigen kit, a sandwich EIA consisting of coated monoclonal antibody (mAb) 12F12 and detection mAb #4, recognizes the E2s domain of *b* HEV but does not bind *r*-1 HEV (15, 16). a.a. sequences of *r* HEV strains were highly divergent from *b* HEV strains in the E2s domain; a human-infecting *r*-1 strain only showed 40.67%–45.33% a.a. identity with *b* HEV (Table 1). Ferret-derived *r*-2 HEV shared 44.67%–48.0% a.a. identity with *b* HEV (Table 1). In contrast, aligning *b*-1 HEV to *b*-4 HEV with *b*-5 HEV to *b*-8 HEV showed 81.76%–93.92% intraspecies a.a. identity (Table S1). We found evolutionary conservation of the E2s domain between *r*-1 HEV and *b*-1 HEV in the top region (black circle) and bottom region (green circle), while the immune-dominant region of the E2s domain located in the groove zone was variable (Fig. 1). The top region of the E2s domain is related to cell receptor binding (28). Evolutionary conservation of the top region indicates why the *r*-1 HEV strain can infect humans. The bottom region of the E2s domain is close to the M domain, distant from the receptor-binding region and immune-dominant region.

**TABLE 1:** Amino acid sequence identities of HEV *ratti* strains compared with HEV *balayani* strains^*b*^

		HEV *balayani* strains							
Viral genotype/GenBank accession number	*B*-1/D11092	*B*-2/M74506	*B*-3/AB369687	*B*-3ra/JX109834	*B*-4/AJ272108	*B*-5/AB573435	*B*-6/AB602441	*B*-7/KT336568	*B*-8/KX387867
	Amino acid identity (%)								
*R*-1/MG813927^*a*^	45.33	44	44	44	42.67	43.33	42.67	42.67	42
*R*-1/MN450851^*a*^	45.33	44	44	44	42.67	43.33	42.67	42.67	42
*R*-1/MN450852^*a*^	45.33	44	44	44	42.67	43.33	42.37	42.37	42
*R*-1/MN450854^*a*^	45.33	44	44	44	42.67	43.33	42.67	42.67	42
*R*-1/MN450853^*a*^	43.33	41.33	42	42.67	40.67	41.33	41.33	40.67	40.67
*R*-1/MK050105^*a*^	44.67	43.33	43.33	44	42	42.67	42	42	41.33
*R*-1/OP610066^*a*^	44	42.67	42.67	42.67	42	42.67	42	41.33	41.33
*R*-2/JN998606	47.33	46.67	47.33	48	46	46	44.67	44.67	45.33

^*a*^ HEV *ratti* strains were reported to be human infected.

^*b*^ *B*, species *balayani; B*-3ra, species *balayani*-3 rabbit strain; HEV, hepatitis E virus; *R*, species *ratti.*

**Fig 2:**
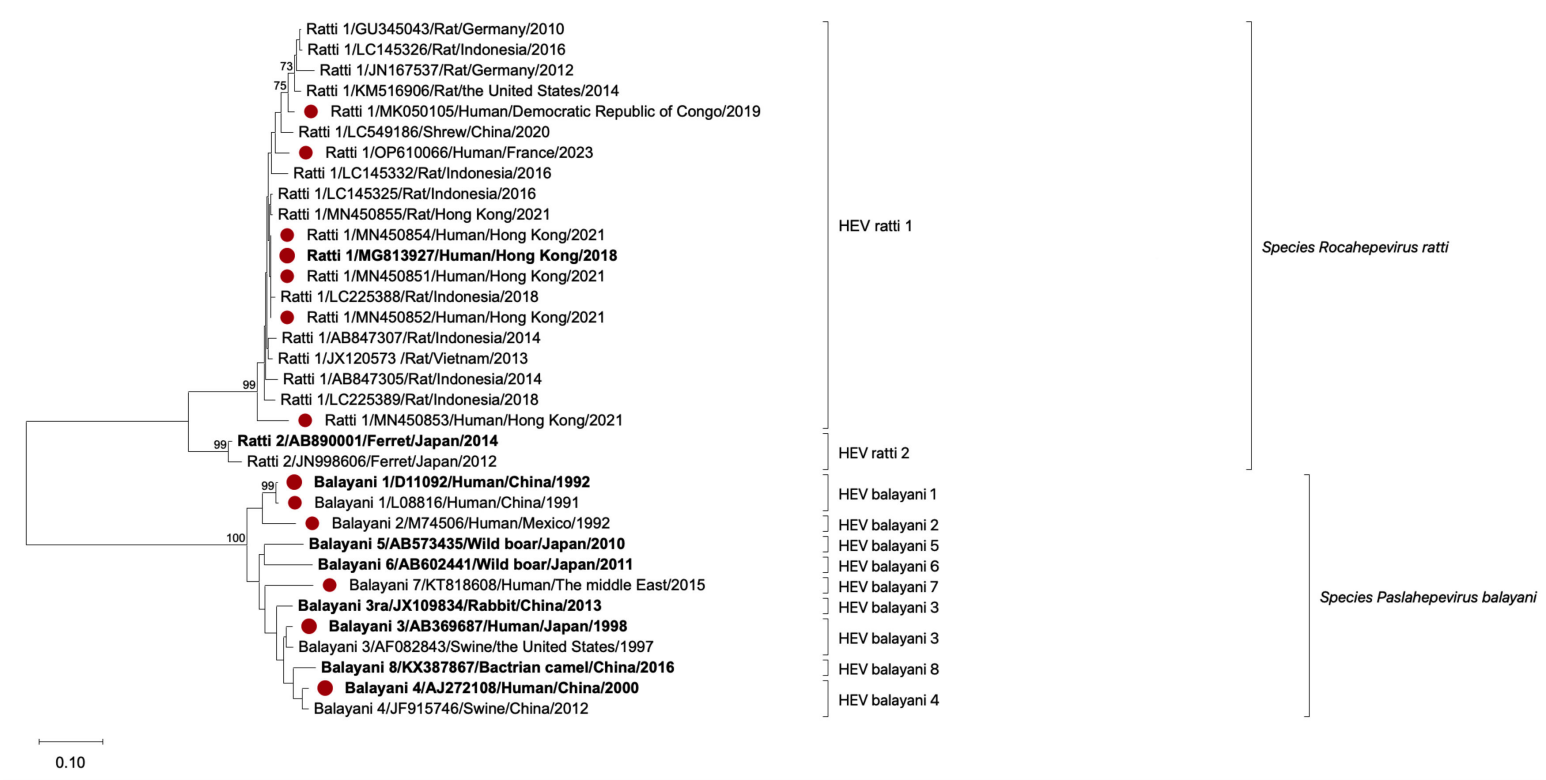
Phylogenetic tree analysis of the HEV a.a. sequence of the E2s domain. Phylogenetic analysis was constructed by the neighbor-joining method based on HEV *balayani* and *ratti* a.a. sequences of the E2s domain. Viral genotype, GenBank accession number, virus host, country of origin, and year of detection are indicated. Sequences obtained from HEV-infected patients are highlighted with a red circle. HEV strains used to express p239 protein in this study are marked in bold. Bootstrap values >70 are shown. Abbreviations: a.a., amino acid.

We analyzed the E2s domain a.a. sequence of 22 human-, rat-, shrew- and ferret-derived *r* HEV strains and compared them with 12 *b* HEV strains by phylogenetic analysis. HEV strains from *r* HEV and *b* HEV formed two scattered subclades. Seven of the human-derived representative *r*-1 HEV strains were from patients in Hong Kong, the Democratic Republic of Congo, and France. Three of the human-derived representative *r*-1 HEV strains (MN450851, MN450852, and MN450854) clustered with the *r*-1 HEV LCK-3110 strain (MG813927) (Fig. 2), which was the first confirmed case of *r*-1 HEV infection. Four *r*-1 HEV strains that infect humans have been reported worldwide. The E2s domain a.a. sequence identity among these strains was 90.67%–96.0% (Table S2). This result indicates that the identity divergence between *r* HEV and *b* HEV strains and among these *r* HEV strains increases the difficulty of antigen assay EIA development.

### Antigen assays for the sandwich EIA

**Fig 3:**
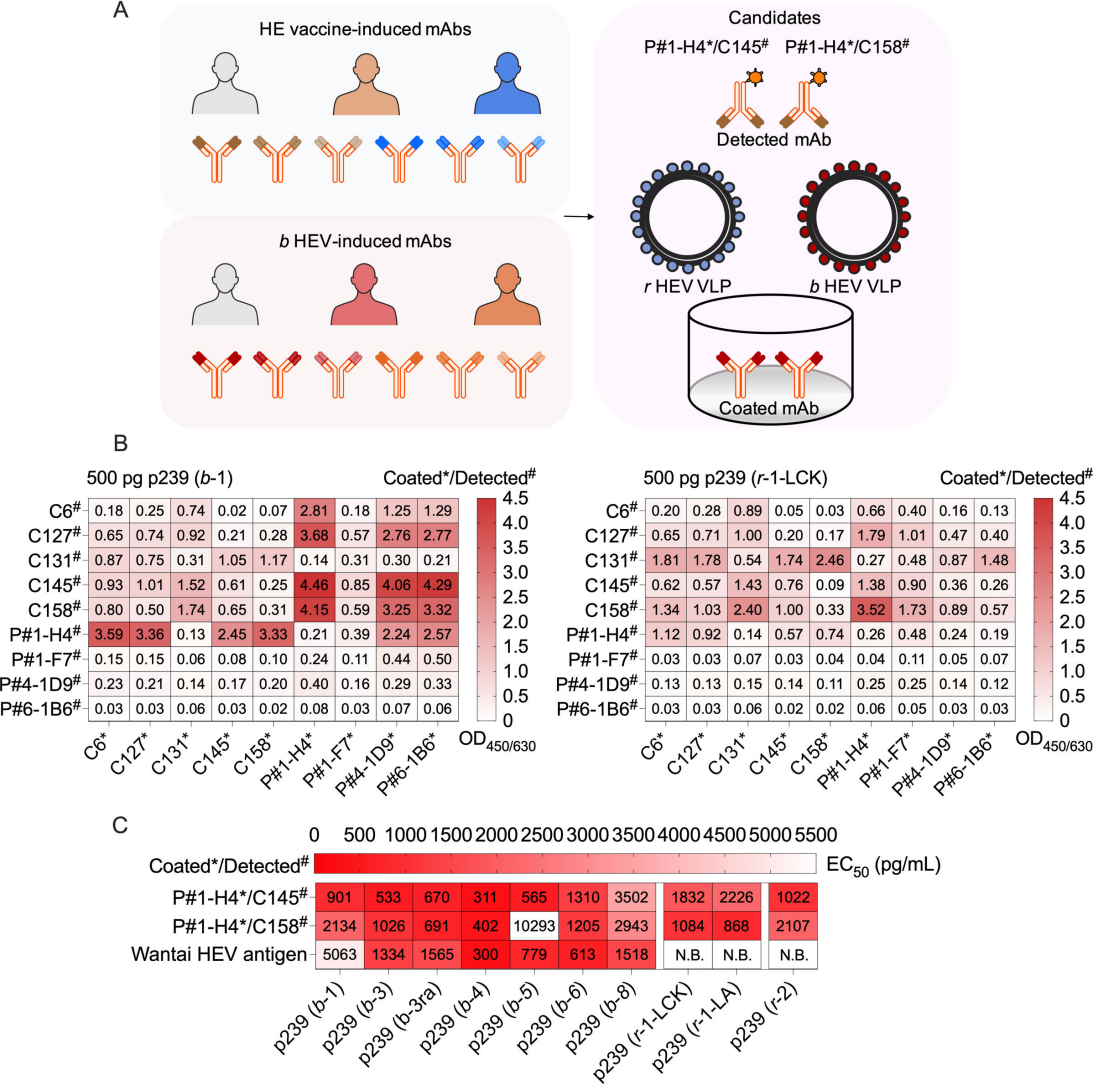
Performance of antigen assays for detecting *r* HEV and *b* HEV antigen. (**A**) Overview of antigen assay workflow. The overall scheme is shown, representing the antigen detection assay evaluation. HE vaccine- and *b* HEV-induced mAbs were collected to separately test binding with *r* HEV and *b* HEV VLPs. Candidates that simultaneously bound with VLPs of two HEV species were enriched from these mAbs and were further used for the sandwich EIA evaluation. (**B**) Preliminary evaluation of the sandwich EIA. Nine candidate mAbs were used to evaluate the performance for detecting VLPs of the *r*-1 HEV LCK 3110 strain and the *b*-1 HEV Xinjiang strain using a 9 × 9 the cross two-mAb sandwich EIA. (**C**) Performance of the candidate sandwich EIA. The performance of two candidate sandwich EIA was evaluated by EC_50_ using serial dilutions of VLP samples from two HEV species. Each VLP sample was tested in duplicate. Abbreviations: *b*-3ra, species HEV *balayani*-3 rabbit strain; *b* HEV, species HEV *balayani*; coated*/detected^#^, coated mAb*/detected mAb^#^; EC_50_, the half-maximal effective concentration; EIA, enzymatic immunoassay; HEV, hepatitis E virus; *r* HEV, species HEV *ratti*; *r-1-LAB*, *species HEV ratti-1 LA-B350 strain*; *r*-1-LCK, species HEV *ratti*-1 LCK-3110 strain; mAb, monoclonal antibody; N.B., nonbinding; VLP, virus-like particle.

Given the divergence of *r* HEV antigenic sites, we hypothesized that conserved sites in the E2s domain between *r* HEV and *b* HEV exist. For antigen assay EIA discovery efforts (Fig. 3A), we collected mAbs from HE-vaccinated and *b* HEV-infected individuals to separately examine binding with *r* HEV and *b* HEV VLPs. Nine mAb candidates that bind simultaneously to VLPs of both HEV species were obtained. To preliminarily assess the performance of antigen assays, paired mAbs were tested in a 9 × 9 matrix in a sandwich EIA format. Most candidates exhibited high binding capacity for detecting 5,000-pg VLP of the *r*-1 HEV LCK-3110 strain and *b*-1 HEV Xinjiang strain, while some sandwich EIA candidates still exhibited low OD values for detecting 50-pg VLP of the two strains (Fig. S1). We selected two-mAb sandwich EIA candidates of OD values of >1 for detecting 500-pg VLP of two strains to further verify performance using serial dilutions of VLPs from the two HEV species (Fig. 3B).

Two mAb pairs, P#1-H4*/C145^#^ and P#1-H4*/C158^#^, exhibited half-maximal effective concentrations (EC_50_) of <2,500 pg/mL for detecting *r*-1 HEV LCK 3110 strain VLP, and their sensitivities tended to be similar between species *r* HEV VLPs and species *b* HEV VLPs, although the EC_50_ of *P*#1-H4*/C158^#^ for detecting *b*-5 HEV VLPs was relatively low (Fig. 3C). The Wantai HEV antigen kit lost the capacity to detect *r* HEV VLPs (Fig. 3C). However, the performance of the remaining mAb pair candidates was significantly worse than that of the two candidates P#1-H4*/C145^#^ and P#1-H4*/C158^#^ for detecting species *r* HEV VLPs and species *b* HEV VLPs (Fig. S2). Collectively, these results confirm that conserved sites recognized by mAbs in the E2s domain exist between *r* HEV and *b* HEV. Two candidates, P#1-H4*/C145^#^ and P#1-H4*/C158^#^, were superior to other mAb pairs for detecting HEV VLPs from both species.

### Performance of the candidate for diagnosis of rat serum samples

**Fig 4:**
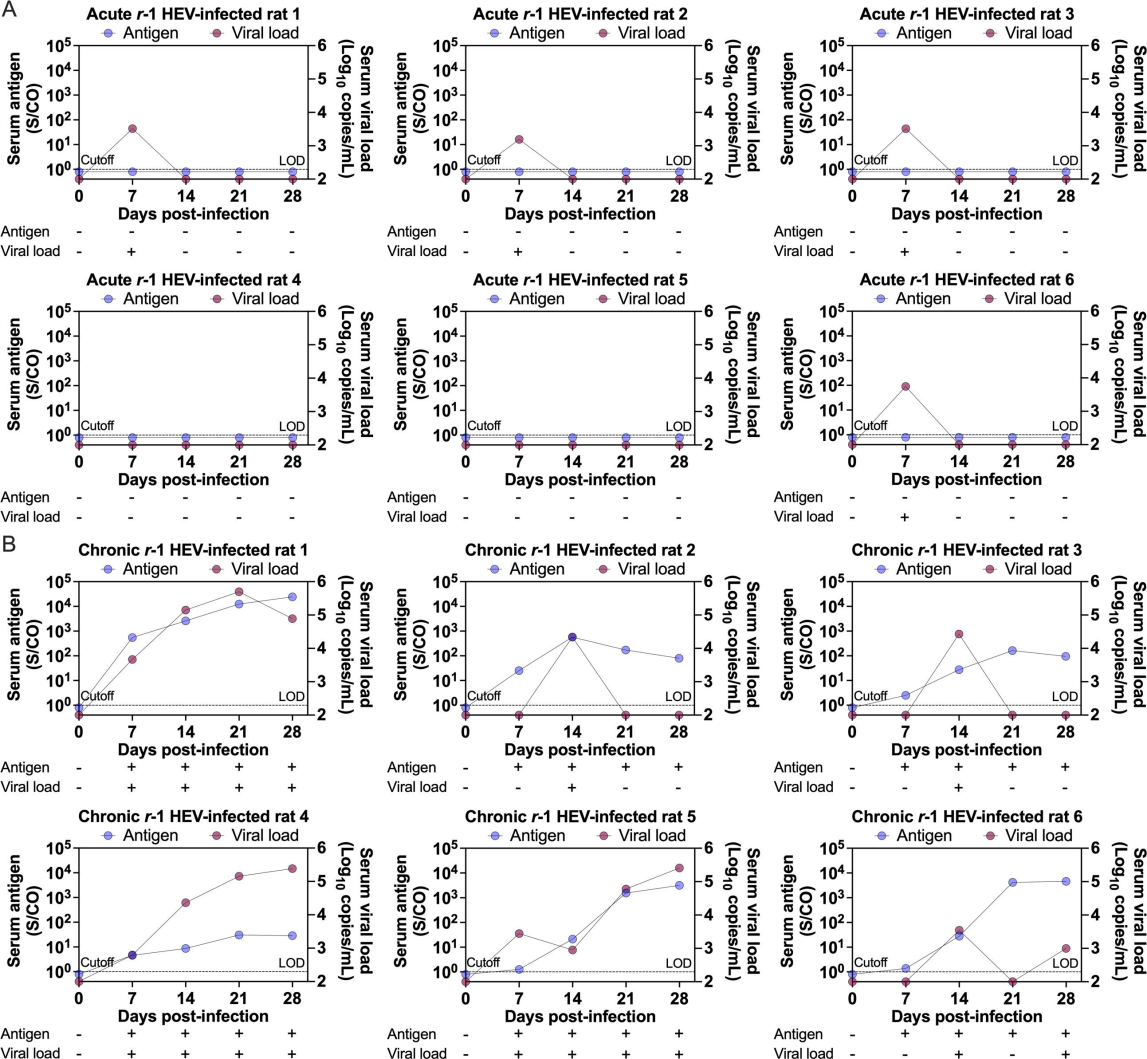
Performance of the antigen assays for diagnosis of *r*-1 HEV-infected rats. The candidate P#1-H4^*^/C158^#^ sandwich EIA was chosen as a representative to evaluate performance for the diagnosis of rat HEV infection. The performance of the EIA assays was evaluated by S/CO using serial dilutions of serum samples from acute (**A**) and chronic (**B**) *r*-1 HEV-infected rats. Dark blue dots represent the antigen assay, and dark red dots indicate nucleic acid testing. The presence and absence of HEV antigen and HEV nucleic acid in sera are indicated by “+” and “–”, respectively. The dashed line indicates the antigen assay cutoff and nucleic acid testing LOD. The candidate sandwich EIA assay cutoff was set to the mean value + 5 SD value in healthy rat controls. Abbreviations: coated*/detected^#^, coated mAb*/detected mAb^#^; EIA, enzymatic immunoassay; HEV, hepatitis E virus; LOD, limit of detection: *r*-1 HEV, *R. ratti* genotype 1; S/CO, signal to cutoff; SD, standard deviation.

To verify the performance for the diagnosis of rat HEV infection, the candidate P#1-H4*/C158^#^ sandwich EIA was chosen as a representative to evaluate rat serum samples. Rat sera collected on days 0, 7, 14, 21, and 28 during *r*-1 HEV infection (*n* = 6 rats per group) were collected as described previously (16, 27). Rats were either immunocompetent, in which case they had self-limiting acute infections, or immunocompromised, in which case they had persistent hepatitis E infections. Rat HEV antigen and HEV real-time RT‒PCR detection was performed in all serum samples. Viral nucleic acid data for these animals were described in our previous publication (27). Four of the six acute *r*-1 HEV-infected rats (66.67%) developed viremia on day 7 with viral loads from 1.55 × 10^3^ copies/mL to 5.58 × 10^3^ copies/mL. All rats cleared viremia on day 14 with no reactivation until day 28. This result reveals that viremia declined and cleared rapidly and that rat HEV infection is self-limiting in immunocompetent rats. None of these rats had detectable antigen in their blood at any timepoint using the P#1-H4*/C158^#^ sandwich EIA (Fig. 4A).

Likewise, we analyzed viremia and antigenemia in chronic rat HEV infection. Due to immunosuppression, four of the six chronic *r*-1 HEV-infected rats (66.67%) had sustained viremia until day 28, with viral loads from 9.95 × 10^2^ copies/mL to 2.56 × 10^5^ copies/mL, and persistent viremia was observed in three of the six chronic *r*-1 HEV-infected rats (50%) (Fig. 4B). Persistent antigenemia was observed in all chronic *r*-1 HEV-infected rats until day 28 by using the candidate P#1-H4*/C158^#^ (Fig. 4B). Detectable antigenemia in chronic *r*-1 HEV-infected rats completely coincided with viremia. In addition, antigenemia also remained during the cleared viremia period. These results indicated that rat HEV antigen was detected by the representative candidate P#1-H4*/C158^#^ sandwich EIA. During the persistent viral replication period, antigenemia detection tended to be superior to viremia detection.

### Performance of the two candidates for diagnosis of clinical serum samples

**Fig 5:**
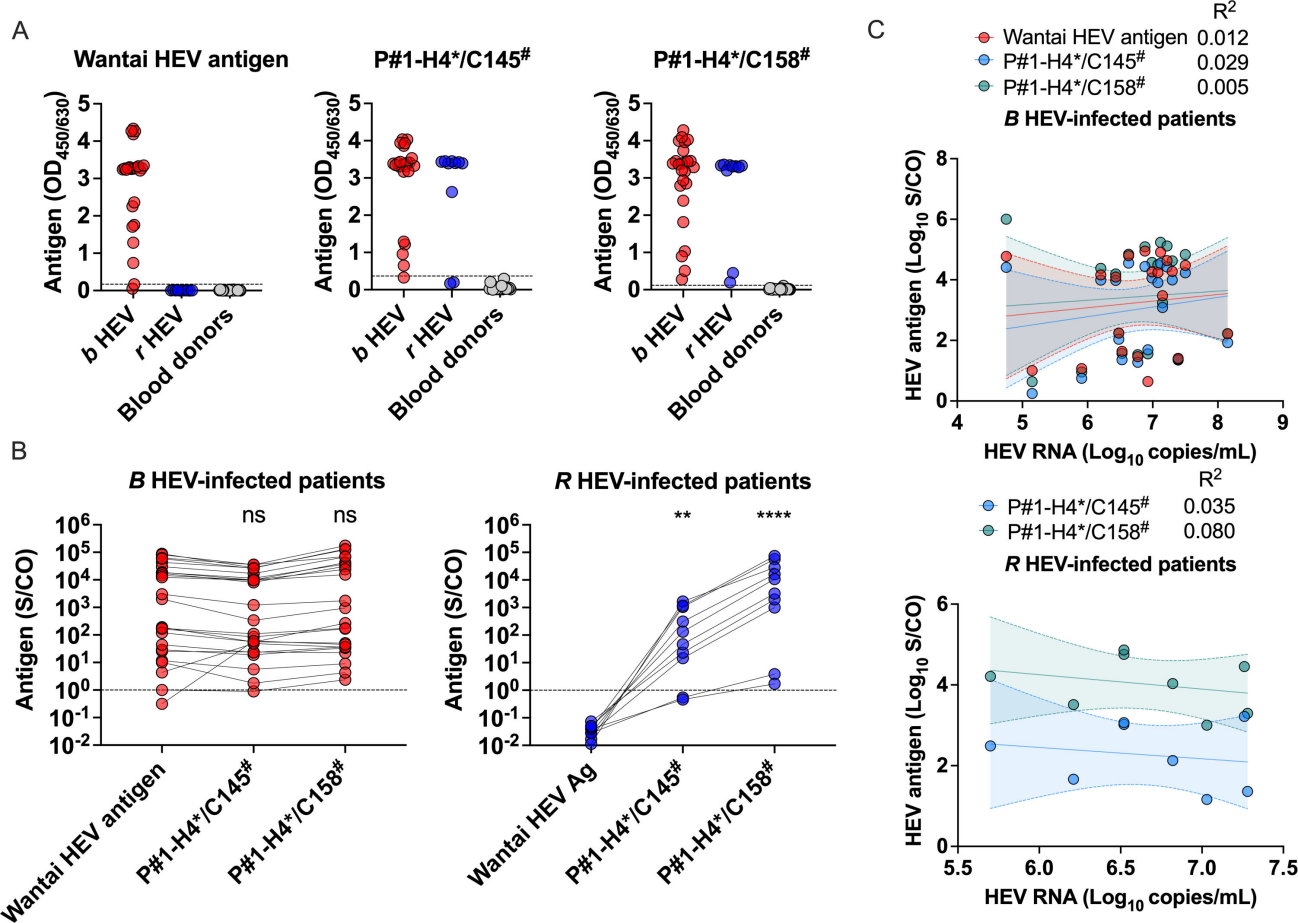
Performance of antigen assays for diagnosis of *r*-1 HEV- and *b* HEV-infected patients. (**A**) OD values of serrum samples in three panels using the Wantai HEV antigen EIA and two candidate sandwich EIAa. Dark red dots in panel A indicate *b* HEV-infected patients; dark blue dots in panel B indicate *r*-1 HEV-infected patients; and dark gray dots in panel C indicate healthy controls. The dashed line indicates the antigen assay cutoff. The Wantai HEV antigen EIA cutoff was set to the standard of the kit, and the two-candidate sandwich EIA assay cutoff was set to the mean value +5 SD value in healthy controls. (**B**) S/CO values of serum samples in two panels using the Wantai HEV antigen EIA and two candidate sandwich EIAs. The performance of three sandwich EIA assays was evaluated by S/CO using serial dilutions of serum samples from two panels. The dashed line indicates the antigen assay cutoff. The mean S/CO of each sandwich EIA assay in each panel was compared to the mean S/CO of the Wantai HEV antigen EIA using the nonparametric Kruskal‒Wallis test with Dunn’s multiple comparisons correction as appropriate. (**C**) Correlation between HEV RNA and HEV antigen in clinical serum samples. The correlation of sandwich EIA assays in two panels was evaluated using a simple linear regression model. The fitted goodness of the model was evaluated by *R* ^2^. The solid line represents the best-fit equation. Bands of 95% CI of each model in two panels are within dashed line. Each serum sample was tested in duplicate. **P < 0.01, ****P < 0.0001. Abbreviations: CI, confidence interval; coated*/detected^#^, coated mAb*/detected mAb^#^; EIA, enzymatic immunoassay; HEV, hepatitis E virus; *b* HEV, species HEV *balayani*; ns, no significance; OD, optical density; *r* HEV, species HEV *ratti*; S/CO, signal to cutoff; SD, standard deviation.

Patient and blood donor serum panels were then analyzed using sandwich EIA candidates. The performances of the commercial Wantai HEV antigen and two sandwich EIA candidates were compared in three panels from 25 *b* HEV-infected patient blood samples (panel A), 15 of whom had immunosuppressive conditions; 10 *r*-1 HEV-infected patient blood samples (panel B), 9 of whom had immunosuppressive conditions; and 27 blood donor blood samples (panel C) (Table 2). The clinical properties and viral load characteristics of *b* HEV and *r*-1 HEV-infected blood samples are listed in Table S3. Panel B comprises 10 of 16 *r*-1 HEV-infected patients reported in Hong Kong to date (8, 11, 12). HEV RT‒PCR detection was performed in all serum samples. Blood donor blood samples (panel C) were used as negative controls. As reported previously (16), all panel B *r*-1 HEV-infected patient blood samples tested negative for the commercial Wantai HEV antigen with OD values similar to those of panel C samples, while 96% (24 of 25) of panel A *b* HEV-infected patient blood samples tested positive (Fig. 5A). The sensitivity of two sandwich EIA candidates, P#1-H4*/C145^#^ (80%, 8 of 10) and *P*#1-H4*/C158^#^ (100%, 10 of 10), was significantly higher than that of the Wantai HEV antigen (0%, 0 of 10), and the sensitivity of the Wantai HEV antigen (96%, 24/25) and those of two sandwich EIA candidates (96%, 24 of 25 and 100%, 25 of 25) for testing panel A *b* HEV-infected patient blood samples were almost the same (Fig. 5A).

**TABLE 2:** Clinical characteristics of blood samples from HEV patients and blood donors^*b*^

Blood samples from *b* HEV-infected patients (panel A, *N* = 25)	
Immunocompetent conditions, *n*/*N* (%)	10/25 (40)
Abnormal ALT	10/25 (40)
Immunosuppressive conditions, *n*/*N* (%)	15/25 (60)
Diffuse large B-cell lymphoma	5/25 (20)
Hematological malignancy	1/25 (4)
Hemopoietic stem cell transplant	5/25 (20)
Solid organ transplant	4/25 (16)
Infecting genotype, *n*/*N* (%)	
*b*-1 HEV	1/25 (4)
*b*-3 HEV	1/25 (4)
*b*-4 HEV	18/25 (72)
*b* HEV, unknown	5/25 (20)
**Blood samples from** ***r*** **-1 HEV-infected patients** ^***a***^ **(panel B,** ***N*** **= 10)**	
Immunocompetent conditions, *n*/*N* (%)	1/10 (10)
Abnormal ALT	1/10 (10)
Immunosuppressive conditions. *n*/*N* (%)	9/10 (90)
Advanced HIV infection	1/10 (10)
Hemopoietic stem cell transplant	1/10 (10)
Solid organ transplant	6/10 (60)
None	1/10 (10)
Infecting strains, *n* (%)	
*r*-1 HEV LCK-3110-like strain	9/10 (90)
*r*-1 HEV MN450853-like strain	1/10 (10)
**Blood samples from blood donors (panel C, *N* = 27)**	
Immunocompetent conditions, *n* (%)	27/27 (100)
Normal ALT	27/27 (100)
Immunosuppressive conditions (n, %)	0/27 (0)

^*a*^ These *r* HEV-infected patients were reported in a previous study.

^*b*^ ALT, alanine transaminase; *b* HEV, species HEV *balayani*; HEV, hepatitis E virus; *r* HEV, species HEV *ratti*.

The performance of three sandwich EIA antigen assays was evaluated by signal to cutoff (S/CO) using serially diluted serum samples from panel A and panel B. The mean S/CO values of panel A *b* HEV-infected patient blood samples using the Wantai HEV antigen did not significantly differ from those of the two sandwich EIA candidates (Fig. 5B). However, the performance of the two sandwich EIA candidates P#1-H4*/C145^#^ (*P* < 0.01) and P#1-H4*/C158^#^ (*P* < 0.0001) was significantly superior to that of the Wantai HEV antigen for detecting *r*-1 HEV infection, and the performance of the candidate P#1-H4*/C158^#^ tended to be superior to that of the candidate P#1-H4*/C145^#^, although differences in S/CO values did not reach statistical significance (Fig. 5B). To evaluate the correlation between HEV RNA and HEV antigen using three sandwich EIA antigen assays for detecting panel A and panel B, a simple linear regression model was fitted. A similar correlation between HEV RNA and HEV antigen was observed between the Wantai HEV antigen EIA and two sandwich EIA candidates for testing blood samples from *b* HEV-infected patients, and the correlation was low (Fig. 5C). Given the lower HEV antigen levels in acute *b* HEV-infected patients than in chronic *b* HEV-infected patients with immunosuppressive conditions (29), we further analyzed the correlation between RNA and antigen levels by dividing the patients into acute and chronic HEV patients to explain why there was a low correlation between RNA and antigen levels. We found a higher correlation between RNA and antigen levels in acute or chronic patients (Fig. S3A and B). This result revealed that the relationship between RNA and antigen levels is different in acute and chronic HEV patients. Compared to similar viral loads in acute HEV patients, higher antigen levels in serum were observed in chronic HEV patients (Fig. S3A and B). This may be because, unlike infectious HEV capsid-associated virion ORF2 (ORF2^C^) with an abundance of viral nucleic acids, the glycosylated dimer forms of ORF2 (ORF2^S^), which is a nonvirion-associated HEV antigen, are the major antigens in the sera of *b* HEV-infected patients (30, 31), and higher levels of ORF2^S^ were produced in chronic HEV patients than in acute patients compared with ORF2^C^. Considering the low correlation in *r* HEV-infected patient samples, a similar situation may also exist (Fig. 5C). As there was only one acute sample from *r* HEV-infected patient samples, the correlation between RNA and antigen levels in acute and chronic *r* HEV-infected patients could not be analyzed separately, and further study should be explored. Overall, excellent performance for the diagnosis of clinical panel serum samples was demonstrated using the two candidates P#1-H4*/C145^#^ and P#1-H4*/C158^#^.

### Mechanism of viral antigen recognition for two candidates

**Fig 6:**
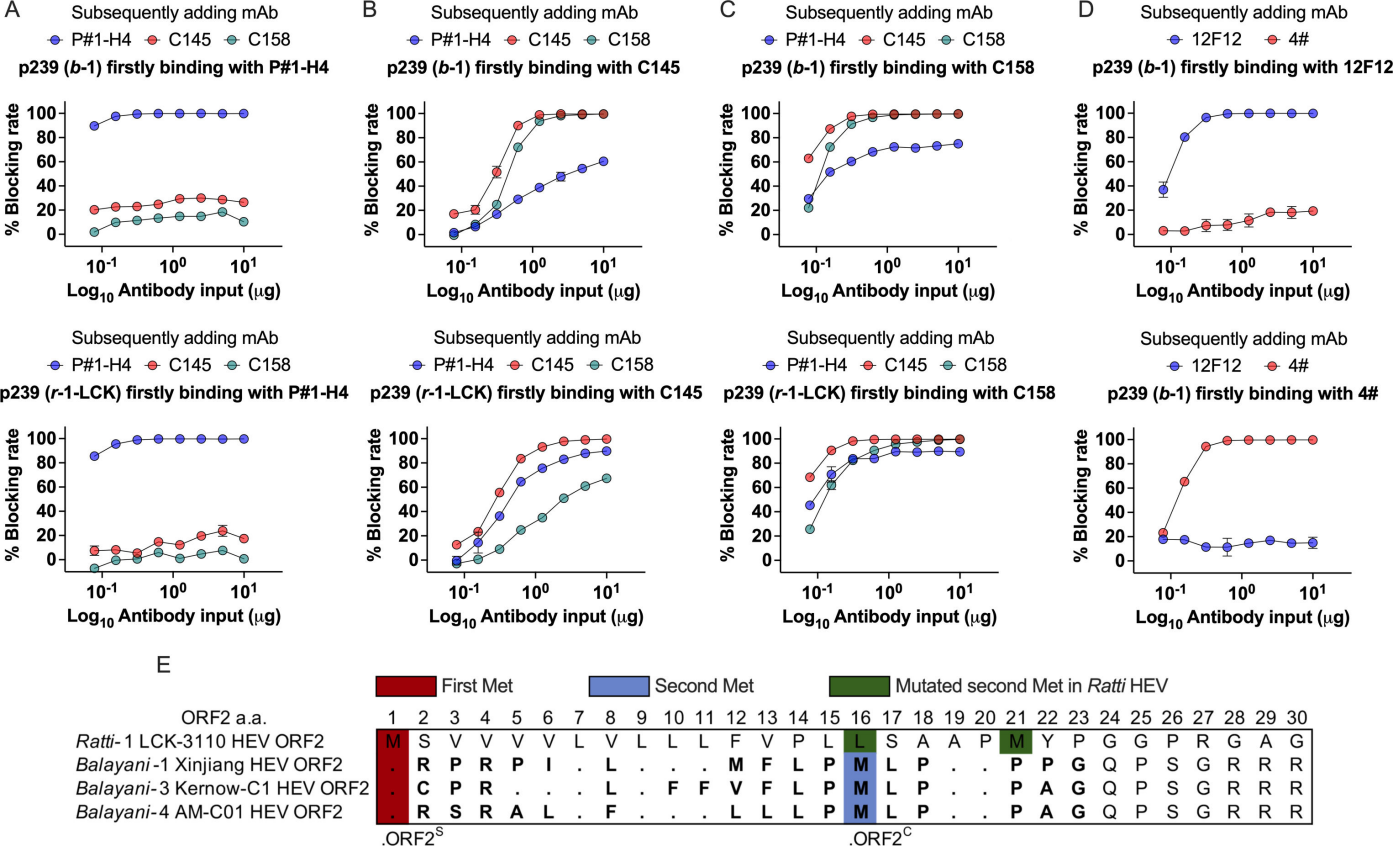
Mechanistic analysis of HEV antigen recognition. The coated mAbs P#1-H4 (**A**), C145 (**B**), C158 (**C**), and 12F12 (**D**) first bound to HEV antigens p239 (*b*-1) and p239 (*r*-1-LCK), respectively. The detected mAbs were subsequently added to the corresponding candidates. The blocking rate of each candidate was calculated according to the control in the presence of PBS. Dots in panels **A–D **represent blocking percentages. Each candidate sample was tested in duplicate. Bars represent the mean and standard error. (**E**) ORF2 sequence alignment of *r*-1, *b*-1, *b*-3, and *b*-4 HEVs. The first Met encoding the secreted form of ORF2 (ORF2^S^) in *b* HEV is shown in red. The internal Met encoding the actual capsid protein of ORF2 (ORF2^C^) in *b* HEV is shown in blue. Different locations of internal Met among the *r*-1 HEV LCK-3110 strain and *b* HEV strain are shown in green. Abbreviations: *b* HEV, species HEV *balayani*; HEV, hepatitis E virus; *r* HEV, species HEV *ratti*; *r*-1-LCK, species HEV *ratti*-1 LCK-3110 strain; M, Met; mAb, monoclonal antibody.

To further explore the mechanism of HEV antigen recognition for the two candidates, we coated p239 (*b*-1) and p239 (*r*-1-LCK) antigens onto the wells of 96-well microplates. Each mAb (P#1-H4, C145, and C158) was first added to bind antigen, and then HEV antigen was detected by subsequently adding HRP-conjugated mAb. P#1-H4 mAb was first added, which hardly blocked the binding of HEV p239 (*b*-1) and p239 (*r*-1-LCK) antigen with C145/C158 mAb (Fig. 6A). In contrast, the C145/C158 mAb was first added to bind antigen, which obviously blocked the binding of antigen with the P#1-H4 mAb, especially with the p239 (*r*-1-LCK) antigen (Fig. 6B and C). These results reveal that P#1-H4 mAb binding to the HEV antigen prior to C145/158 mAb may be beneficial to C145/158 mAb binding, which is a mechanism different from that of the Wantai antigen kit comprising mAbs 12F12 and 4# (Fig. 6D). In addition, we hypothesized that ORF2^S^ is also the major antigen at high levels in the sera of *r-1* HEV-infected patients and analyzed the sequence of *ORF2*. We found that the first Met encoding ORF2^S^ and the internal Met encoding ORF2^C^ exist in ORF2 among the *r*-1 HEV LCK-3110 strain and *b* HEV strain (Fig. 6E), whereas the highly conserved internal Met^16^ among the *b* HEV strain is mutated and transferred to Met^21^ in the *r*-1 HEV LCK-3110 strain. This transformation of internal Met in ORF2 of the *r*-1 HEV LCK-3110 strain did not appear to interfere with the encoding signal peptide of the first Met that could direct the protein into the secretory pathway (Fig. S4). ORF2^S^ at high levels exists in the sera of *r*-1 HEV-infected patients, which may also explain the mechanism by which the ORF2^S^ antigen of *r*-1 HEV is recognized by two EIA candidates.

## DISCUSSION

Rats are the natural reservoirs of *r*-1 HEV (32). In the past, *r*-1 HEV was not considered a zoonosis because of the differential nucleotide homology (identity 50%–60%) between *r*-1 HEV and *b* HEV (32, 33). However, over the past 5 years there have been several cases of zoonotic crossover to humans, with evidence for *r*-1 HEV infection among patients in Hong Kong (8, 11, 12, 34), Central Africa, and Europe (9, 10, 13). As of the time of writing, 21 *r*-1 HEV-human infections have been reported worldwide (16 in Hong Kong, 1 in Central Africa, and 4 in Europe). Lack of awareness of *r*-1 HEV as a cause of hepatitis and unavailability of diagnostics has undoubtedly impaired surveillance efforts for this pathogen. Our two antigen assay candidates P#1-H4*/C145^#^ and P#1-H4*/C158^#^ provide an effective and convenient approach to diagnose and differentiate *r*-1 HEV infection status if paired with the Wantai antigen assay testing. An efficient HEV antibody EIA approach for serodiagnosis is also required to investigate the seroprevalence of *r*-1 HEV infection, as described recently (35).

In the present study, we sought to identify mAbs induced by the HE vaccine and *b* HEV infection that recognized conserved sites in the E2s domain of *r* HEV and *b* HEV for diagnosing *r*-1 HEV infections. Two sandwich EIA candidates, P#1-H4*/C145^#^ and P#1-H4*/C158^#^, were obtained from nine mAb candidates that simultaneously bound to VLPs derived from both species. The P#1-H4*/C145^#^ and P#1-H4*/C158^#^ antigen EIA assays had excellent performance for detecting *r* HEV infection and *b* HEV infection and 10- to 100-pg sensitivity for detecting recombinant HEV VLPs. Notably, ORF2^S^ is the major antigen at high levels in the sera of *b* HEV-infected patients, and the sensitivity according to the detection of recombinant HEV VLPs (ORF2^C^) may be physiologically irrelevant in infected patients.

Phylogenetic tree analysis revealed that, presently, human-derived representative *r*-1 HEV strains are divided into four distinct subclades (MG813927; LCK-3110 strain, MK050105, OP610066, and MN450853), revealing the impact of divergent *r* HEV strains on diagnosis; Spanish *r*-1 HEV strains (OK082152–OK082154) were not included in the phylogenetic tree analysis due to the lack of *ORF2* sequence. We found that two blood samples from *r*-1 HEV-infected patients, C9 infected by the LCK-3110 strain and C18 infected by the MN450853-like strain (Table S3), were more difficult to analyze using the two sandwich EIA candidates P#1-H4*/C145^#^ and P#1-H4*/C158^#^ (Fig. 5A). Blood sample C9 was obtained from a patient with acute *r*-1 HEV infection approximately 2 weeks after symptom onset, and the antigen levels may have been declining while free HEV RNA levels in the serum were still high, reaching 7.2 log_10_ copies/mL. Blood sample C18 came from a patient with *r*-1 HEV MN450853-like strain chronic infection. This strain belongs to a different clade than LCK-3110-like strains (Fig. 2), and the divergence of *r* HEV antigenic sites in the E2s domain may contribute to detection escape. We also noted that C18 was not immunosuppressed and had a strong antibody response to both HEV IgM and IgG (Table S3) (11). Residual HEV particles in sera may have already been bound by anti-HEV IgG, affecting the performance of binding to particles in two sandwich EIA candidates, as previously reported (36).

There were several limitations in this study. First, although blood samples from *r*-1 HEV strain-infected patients in Hong Kong were included in panel B, we were unable to obtain *r*-1 human infection samples from Central African and European cases. HEV strains used to infect rats are phylogenetically closer to European and Central African strains, and rat sera validated the antigen assays for divergent *r*-1 HEV strains. As new patients emerge through surveillance and effective methods for rat HEV diagnosis, more samples will be available to contribute to the optimization of the sandwich EIA assay. Second, a low correlation between HEV RNA and HEV antigen was found in serum samples in both the Wantai antigen assay and the two evaluated antigen assay candidates. In addition to the effect of anti-HEV IgG in serum samples, we found that the first Met encoding the secreted form of the ORF2^S^ protein was conserved among the *r*-1 HEV LCK-3110 strain and the *b* HEV strain (Fig. 5E). Signal peptide sequence exists in the addition of a.a. to the N-terminus ORF2 of *r*-1 HEV LCK-3110 strain via SignalP-6.0 prediction (Fig. S4). Compared to similar viral loads in acute *b* HEV patients, higher antigen levels in serum were observed in chronic *b* HEV patients, causing lower HEV antigen levels in acute *b* HEV-infected patients than in chronic *b* HEV-infected patients (Fig. S3A and B) (29). We hypothesized that ORF2^S^ without viral nucleic acid also exists in the sera of *r*-1 HEV-infected cases (28, 31). However, a lack of serum samples with sufficient volume from *r*-1 HEV-infected cases meant that we could not perform a density gradient separation experiment to verify the existence of ORF2^S^. Further work is necessary to demonstrate this hypothesis. In *b* HEV-infected cases, ORF2^S^ antigen in serum is able to enter urine, and the levels of urine antigen are higher than those of serum antigen (37). A similar phenomenon in urine from *r* HEV-infected patients also requires study.

In summary, HEV antigen assay candidates P#1-H4*/C145^#^ and P#1-H4*/C158^#^ bound to discrete antigenic sites that are highly conserved across *r* HEV and *b* HEV. The candidates have excellent performance for the diagnosis of *r*-1 HEV- and *b* HEV-infected clinical serum samples. We redeveloped antigen detection to solve the missed diagnosis of rat HEV.

## SUPPLEMENTAL MATERIAL

Figures S1 to S4, Tables S1 to S3. jcm.00710-23-s0001.docx Supplementary information

ASM does not own the copyrights to Supplemental Material that may be linked to, or accessed through, an article. The authors have granted ASM a non-exclusive, world-wide license to publish the Supplemental Material files. Please contact the corresponding author directly for reuse.
