# Supplementary material for: Redeveloping antigen detection kits for the diagnosis of rat hepatitis E virus
Source: J Clin Microbiol. 2023 Dec 1;61(12):e00710-23. doi: 10.1128/jcm.00710-23 (PMC10729709; doi:10.1128/jcm.00710-23)
Supplement: Figures S1 to S4, Tables S1 to S3. — Supplementary information [file jcm.00710-23-s0001.docx]

Table S1. Amino acid sequence identities of HEV *balayani* strains.

|  | HEV *balayani* strains | | | | |
| --- | --- | --- | --- | --- | --- |
| Viral genotype/GenBank accession number | *B*-1/  D11092* | *B*-2/  M74506* | *B*-3/  AB369687* | *B*-3ra/  JX109834 | *B*-4/  AJ272108* |
|  | Amino acid identity (%) | | | | |
| *B*-5/AB573435 | 84.46 | 81.76 | 89.19 | 87.84 | 89.86 |
| *B*-6/AB602441 | 83.78 | 81.76 | 87.84 | 86.49 | 89.86 |
| *B*-7/KT336568* | 89.19 | 87.16 | 89.86 | 87.16 | 86.49 |
| *B*-8/KX387867 | 88.51 | 86.49 | 93.92 | 91.22 | 93.92 |

*HEV *balayani* strains were reported to be human-infected.

HEV, hepatitis E virus

*B*, species *balayani*

*B*-3ra, species *balayani*-3 rabbit strain

Table S2. Amino acid sequence identities of HEV *ratti* strains.

|  | HEV *ratti* strains | | | |
| --- | --- | --- | --- | --- |
| Viral genotype/GenBank accession number | *R*-1/  MG813927* | *R*-1/  MN450853* | *R*-1/  MK050105* | *R*-1/  OP610066* |
|  | Amino acid identity (%) | | | |
| *R*-1/MG813927* | 100 | 93.33 | 95.33 | 96 |
| *R*-1/MN450853* | 93.33 | 100 | 92 | 90.67 |
| *R*-1/MK050105* | 95.33 | 92 | 100 | 94.67 |
| *R*-1/OP610066* | 96 | 90.67 | 94.67 | 100 |

*HEV *ratti* strains were reported to be human-infected.

HEV, hepatitis E virus

*R*, species *ratti*

Table S3. Clinical properties and viral load characteristics of *b* HEV and *r*-1 HEV blood samples.

| **Blood samples from *b* HEV-infected patients (Panel A, n = 25)** | | | |
| --- | --- | --- | --- |
| Patient | Immunosuppressive condition | Type of infection | *b* HEV viral load (log_10_ copies/mL) |
| AM-C01 AA | Hemopoietic stem cell transplant | Chronic |  |
| Serial sample code (n = 4) |  |  |  |
| AA (-97) |  |  | 6.88 |
| AA (-7) |  |  | 7.12 |
| AA (36) |  |  | Negative |
| AA (103) |  |  | Negative |
| Patient | Immunosuppressive condition | Type of infection | *b* HEV viral load (log_10_ copies/mL) |
| AM-C02 DLBCL | DLBCL | Chronic |  |
| Serial sample code (n = 5) |  |  |  |
| DLBCL-1 |  |  | 7.22 |
| DLBCL-2 |  |  | 7.50 |
| DLBCL-3 |  |  | 6.99 |
| DLBCL-4 |  |  | 7.30 |
| DLBCL-5 |  |  | 7.08 |
| Patients | Immunosuppressive condition | Type of infection | *b* HEV viral load (log_10_ copies/mL) |
| Other | None | Acute |  |
| Sample code  (n = 16) |  |  |  |
| JHY |  |  | 5.91 |
| HNZZ |  |  | 8.15 |
| 496 |  |  | Negative |
| 553 |  |  | 7.39 |
| 616 |  |  | 6.77 |
| 677 |  |  | 6.48 |
| 679 |  |  | 6.53 |
| 852 |  |  | 7.15 |
| Human 3 |  |  | 6.93 |
| Human 4 |  |  | 6.18 |
| Human 5 | Hemopoietic stem cell transplant |  | 7.59 |
| Human 6 | Solid organ transplant | Chronic | 6.20 |
| Human 7 | Solid organ transplant |  | 5.15 |
| Human 8 | Solid organ transplant |  | 6.63 |
| Human 9 | Solid organ transplant |  | 6.43 |
| Human 10 | Hemopoietic stem cell transplant |  | 4.75 |
| **Blood samples from *r*-1 HEV-infected patients (Panel B, n = 10)** | | | |
| Sample code  (n = 10) | Immunosuppressive condition | Type of infection | *r*-1 HEV viral load (log_10_ copies/mL) |
| C3 | Solid organ transplant | Chronic | 7.03 |
| C5 | Solid organ transplant | Chronic | 6.52 |
| C8 | Advanced HIV infection | Chronic | 7.26 |
| C9 | None | Acute | 7.20 |
| C15 | Hemopoietic stem cell transplant | Chronic | 7.28 |
| C16 | Solid organ transplant | Chronic | 5.70 |
| C18 | None | Chronic | 6.37 |
| C23 | Solid organ transplant | Chronic | 6.52 |
| C24 | Solid organ transplant | Chronic | 6.82 |
| C27 | Solid organ transplant | Chronic | 6.21 |

AA, aplastic anemia

DLBCL, diffuse large B-cell lymphoma


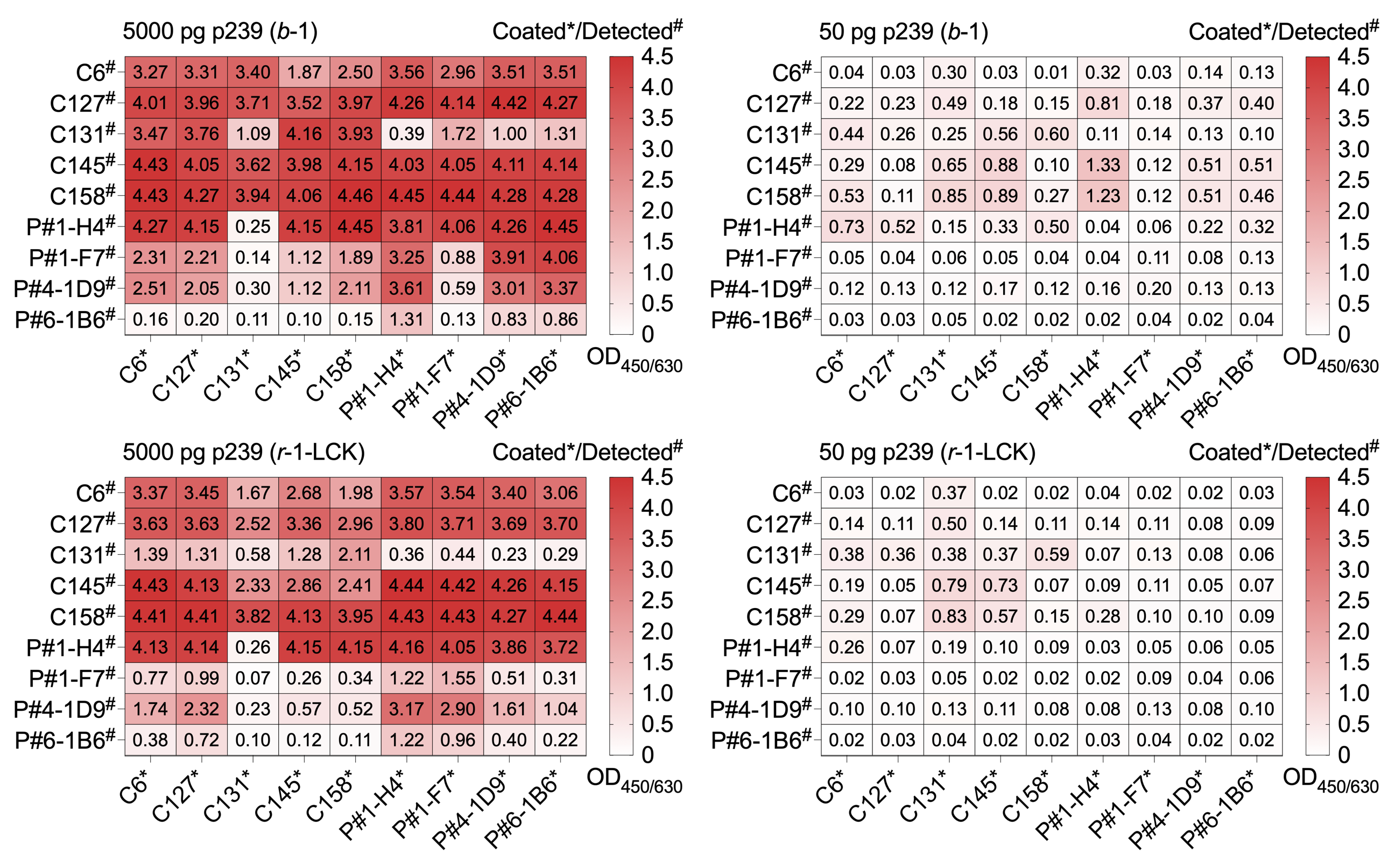


**Figure S1.** **Preliminary evaluation of the sandwich EIA.** Nine candidate mAbs were used for evaluating performance for detecting VLPs of *r*-1 HEV LCK 3110 strain and *b*-1 HEV Xinjiang strain using a 9 × 9 the cross two-mAb sandwich EIA. Abbreviations: Coated*/Detected^#^, coated mAb*/detected mAb^#^; EIA, enzymatic immunoassay; HEV, hepatitis E virus; *b* HEV, species HEV *balayani*; *r* HEV, species HEV *ratti*; mAb, monoclonal antibody; VLPs, virus-like particles.


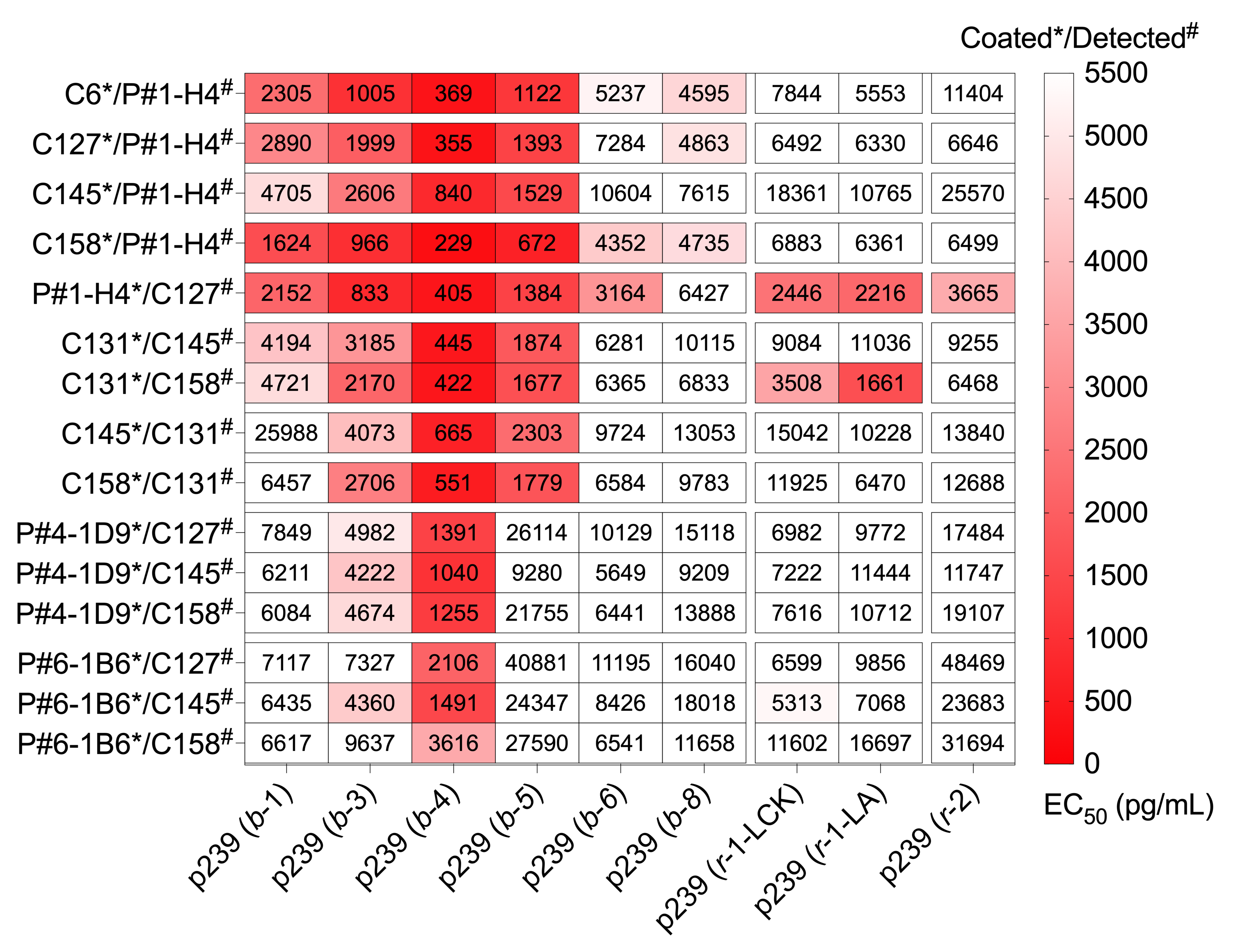


Figure S2. Performance of the candidate sandwich EIA. Performance of candidate sandwich EIA was evaluated by EC_50_ using serial dilutions of VLP samples from two HEV species. Abbreviations: Coated*/Detected^#^, coated mAb*/detected mAb^#^; EC_50_, the half-maximal effective concentration; EIA, enzymatic immunoassay; HEV, hepatitis E virus; *r*-1-LCK, species HEV *ratti*-1 LCK-3110 strain; *r*-1-LAB, species HEV *ratti*-1 LA-B350 strain; VLP, virus-like particle.


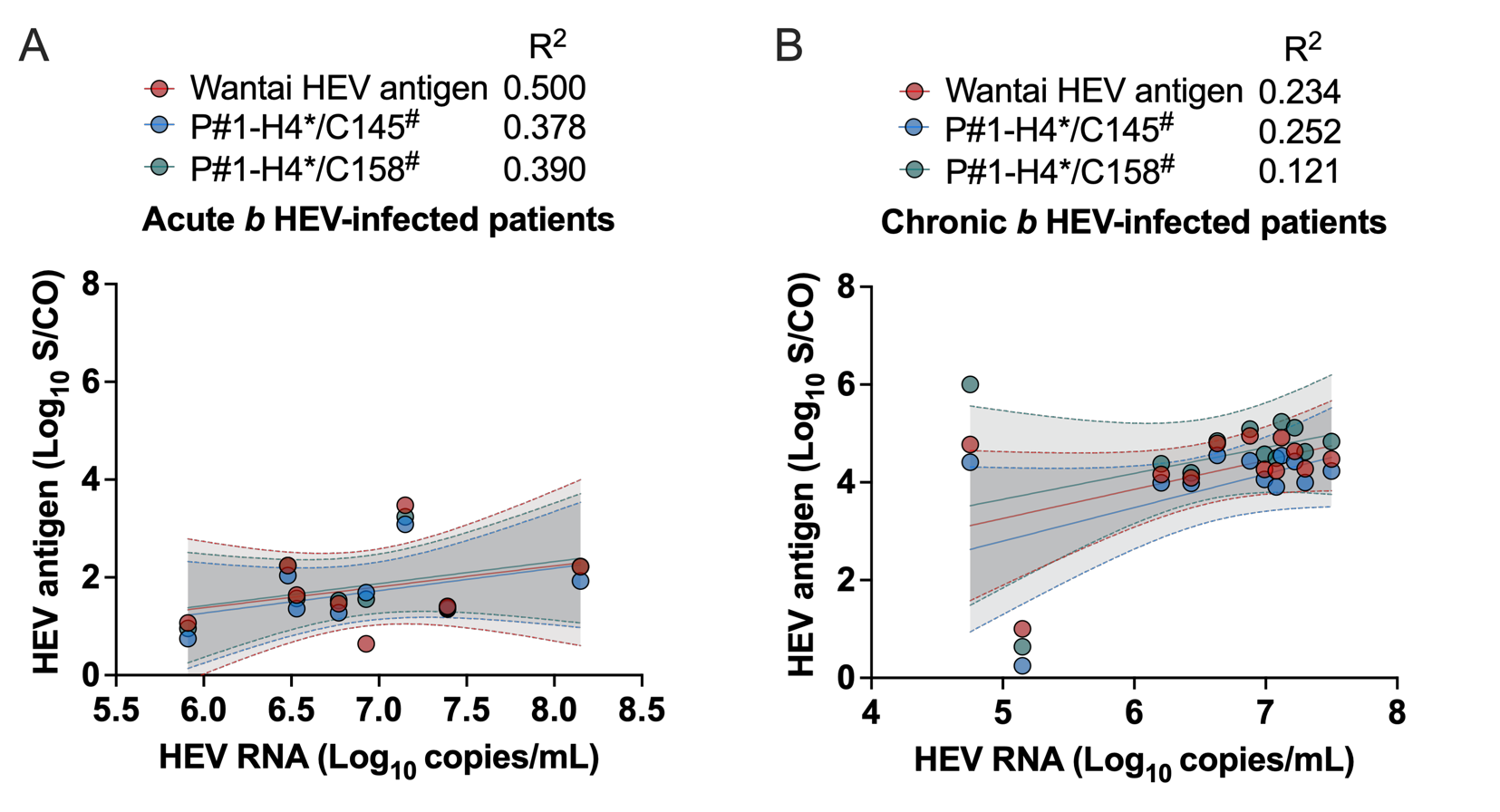


**Figure S3. Correlation between HEV RNA and HEV antigen.** Correlation between HEV RNA and HEV antigen in clinical sera samples from acute (A) and chronic (B) *b* HEV-infected patients. Correlation of sandwich EIA assays in *b* HEV-infected patients was evaluated using simple linear regression model. Fitted goodness of the model was evaluated by R^2^. Solid line represents best-fit equation. Bands of 95% CI of each model in two panels are within dashed line. Each sera sample was tested in duplicate. Abbreviations: CI, confidence interval; Coated*/Detected^#^, coated mAb*/detected mAb^#^; EIA, enzymatic immunoassay; HEV, hepatitis E virus; *b* HEV, species HEV *balayani*; S/CO, signal to cutoff.


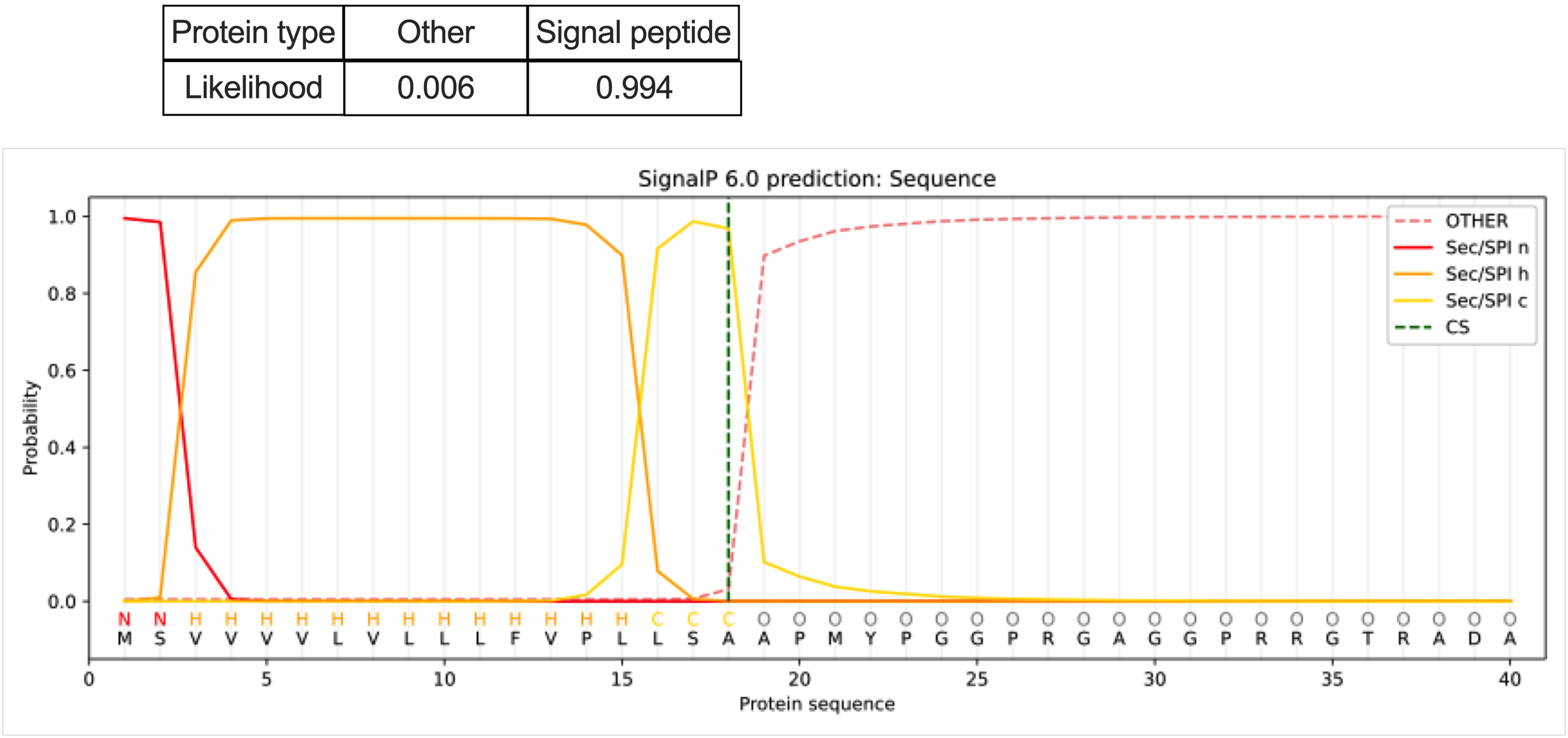


Figure S4. Prediction of signal peptide in *r*-1 HEV LCK-3110 strain ORF2. The SignalP 6.0 server predicts the presence of signal peptides and the location of their cleavage sites in proteins. The first 40 a.a. to the N terminus ORF2 of *r*-1 HEV LCK-3110 strain was submitted in The SignalP 6.0 server for signal peptide prediction.
